# Supplementary material for: Ectopic Activation of Fgf8 in Dental Mesenchyme Causes Incisor Agenesis and Molar Microdontia
Source: Int J Mol Sci. 2024 Jun 27;25(13):7045. doi: 10.3390/ijms25137045 (PMC11241644; doi:10.3390/ijms25137045)
Supplement: Supplementary file 1 [file ijms-25-07045-s001.zip › Supplementary.docx]

**Supplementary materials**

**Figure S1.** The regressed maxillary incisor germs of *Osr2-cre^KI^;Rosa26R-Fgf8* mice. The Masson staining of E14.5 WT (A) and *Osr2-cre^KI^;Rosa26R-Fgf8* maxillary incisors (**A’**), E15.5 WT (**B**) and*Osr2-cre^KI^;Rosa26R-Fgf8*maxillary incisors (**B’**), E16.5 WT (**C**) and *Osr2-cre^KI^;Rosa26R-Fgf8* maxillary incisors (**C’**), and E18.5 WT (**D**) and *Osr2-cre^KI^; Rosa26R-Fgf8* maxillary incisors (**D’**). The yellow dashed lines contoured enamel organs in incisor germs. Scale bar, 200 μm.

**
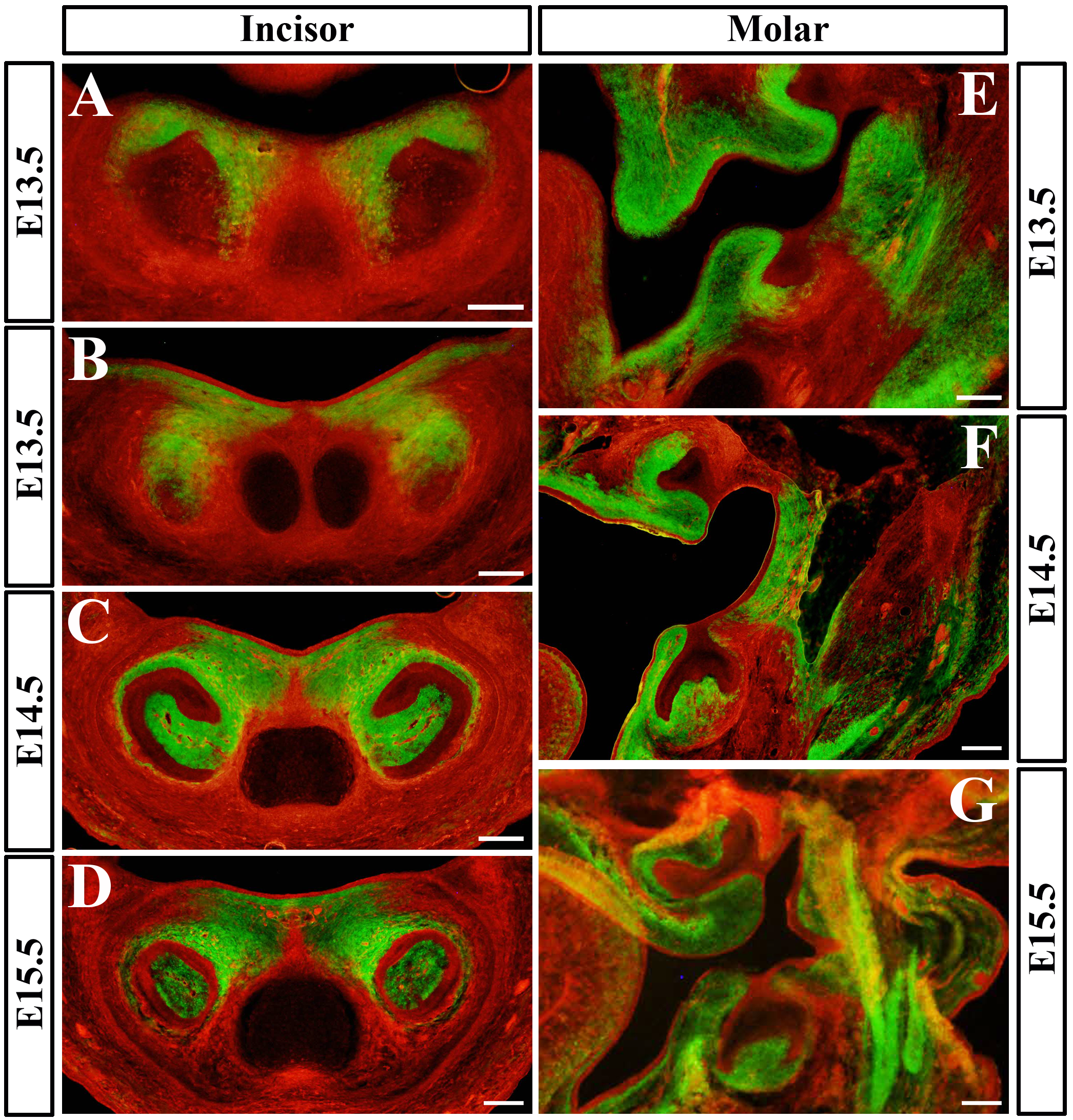
***8* maxillary incisors (**A’**), E15.5 WT (**B**) and*Osr2-cre^KI^;Rosa26R-Fgf8*maxillary incisors (**B’**), E16.5 WT (**C**) and *Osr2-cre^KI^;Rosa26R-Fgf8* maxillary incisors (**C’**), and E18.5 WT (**D**) and *Osr2-cre^KI^; Rosa26R-Fgf8* maxillary incisors (**D’**). The yellow dashed lines contoured enamel organs in incisor germs. Scale bar, 200 μm.

**
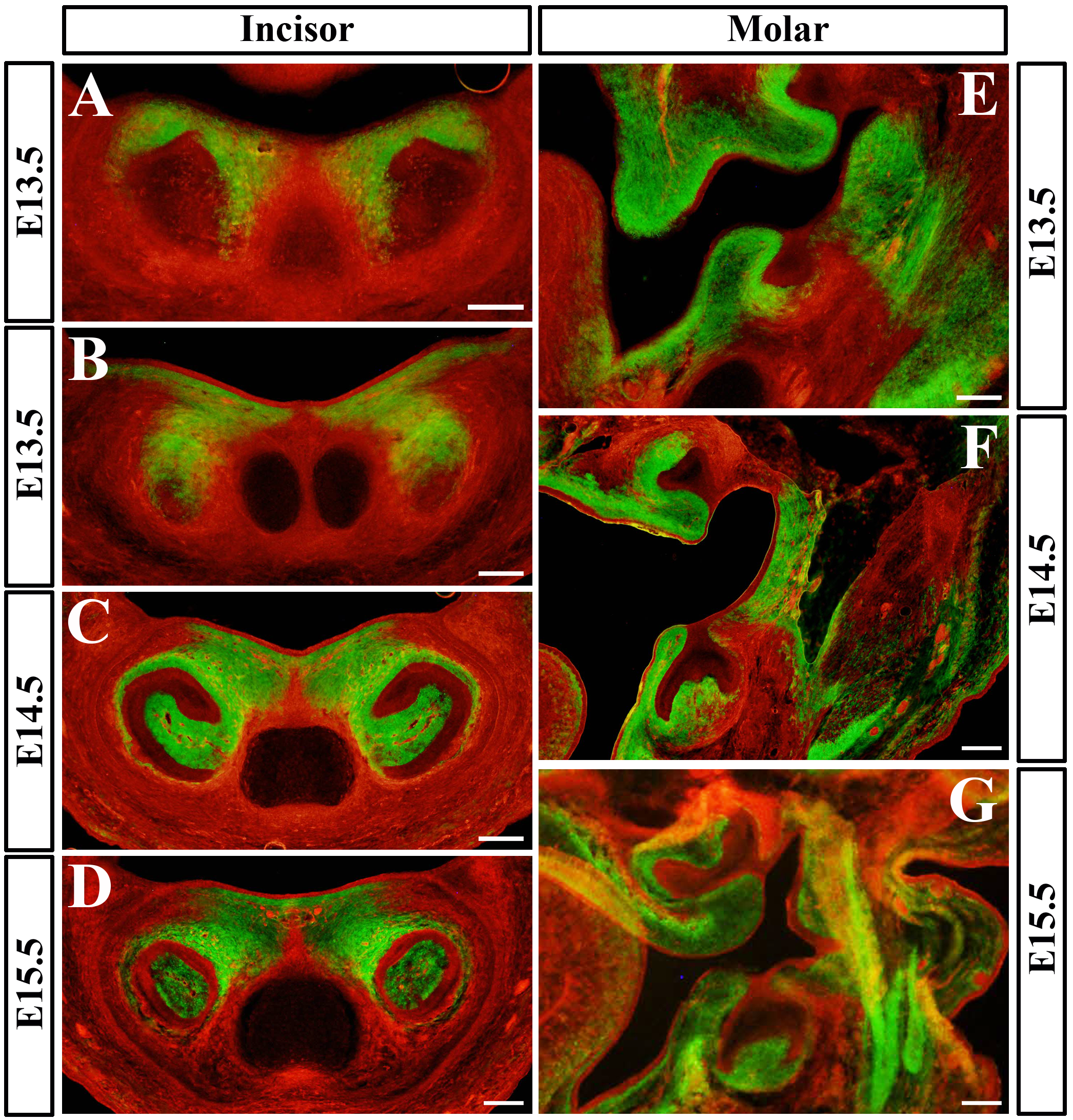
Figure S2.** The Cre pattern in *Osr2-cre^KI^;Rosa26R-mT/mG* incisor and molar germs. The cryostat sections of the anterior (**A**) and posterior (**B**) E13.5 *Osr2-cre^KI^;Rosa26R-mT/mG* incisor germs, E14.5 *Osr2-cre^KI^;Rosa26R-mT/mG* incisor germs (**C**), and E15.5 *Osr2-creKI; Rosa26R-mT/mG* incisor germs (**D**). The cryostat sections of E13.5 *Osr2-cre^KI^; Rosa26R-mT/mG* molar germs (**E**), E14.5 *Osr2-cre^KI^;Rosa26R-mT/mG* molar germs (F), and E15.5 *Osr2-cre^KI^;Rosa26R-mT/mG* and molar germs (**G**). Scale bar, 200 μm.
