# Supplementary figures and images for: Ectopic Activation of Fgf8 in Dental Mesenchyme Causes Incisor Agenesis and Molar Microdontia
Source: Int J Mol Sci. 2024 Jun 27;25(13):7045. doi: 10.3390/ijms25137045 (PMC11241644; doi:10.3390/ijms25137045)

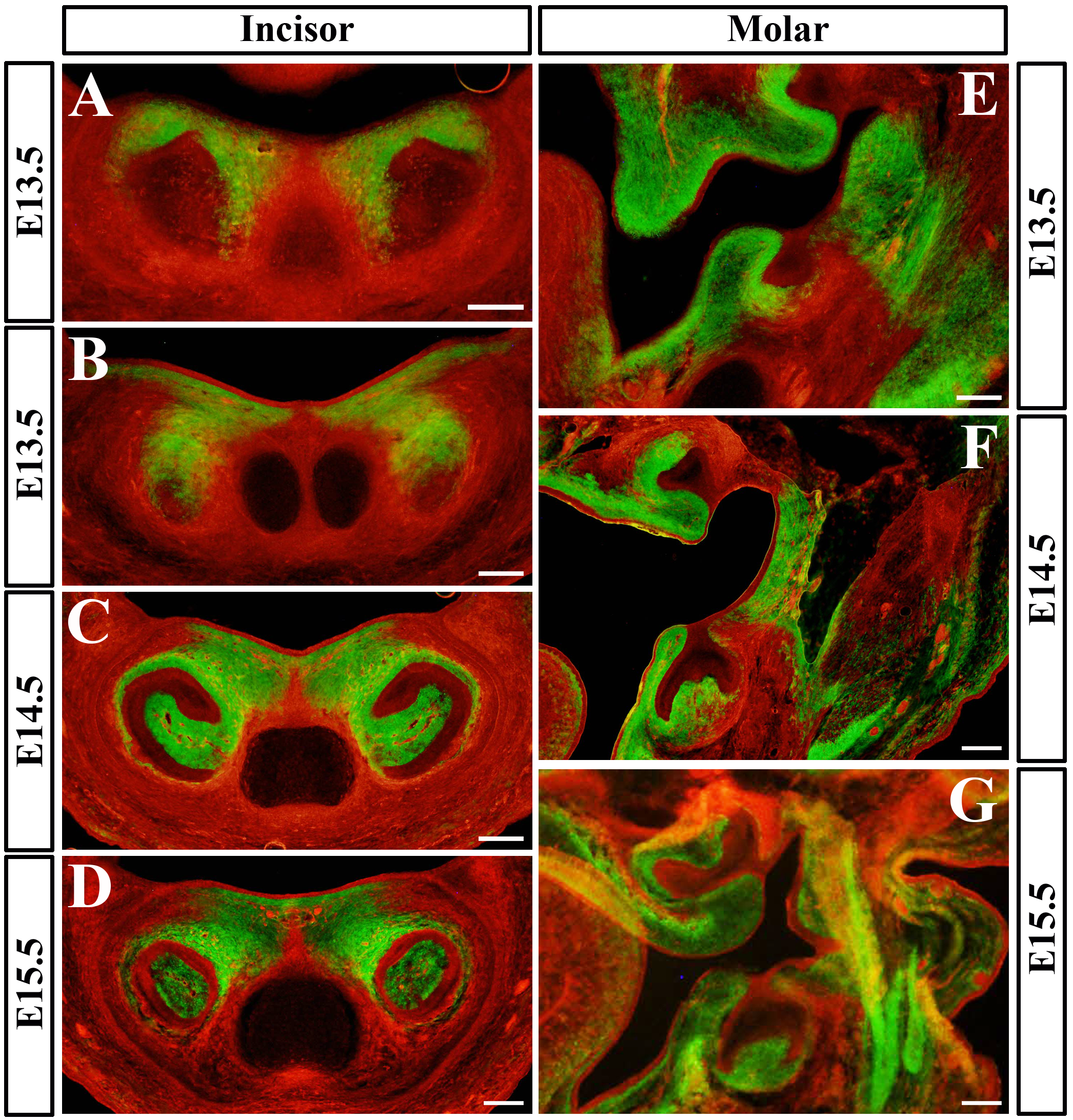

Supplement: Supplementary file 1 [file ijms-25-07045-s001.zip › Fig S2.tif]

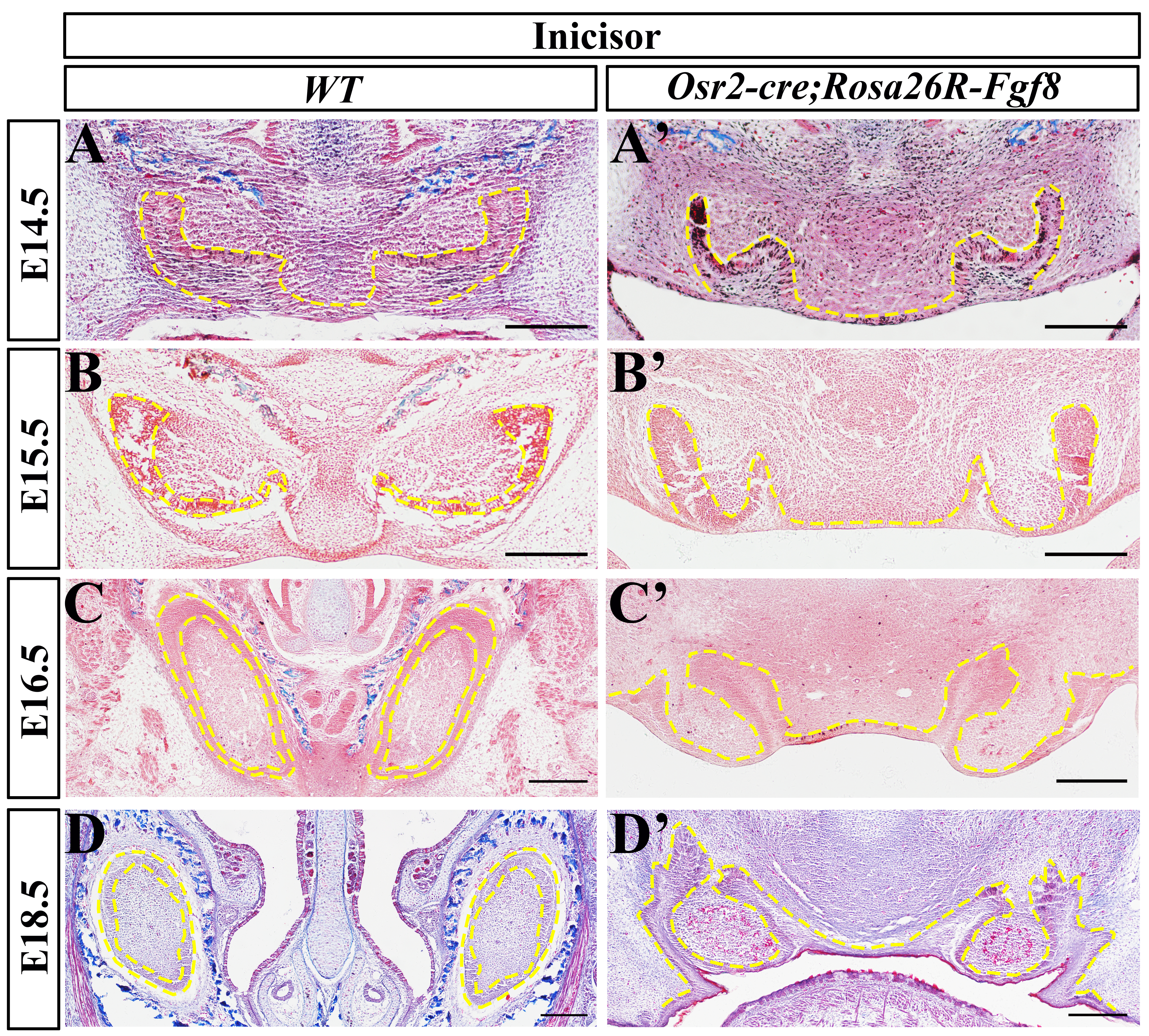

Supplement: Supplementary file 1 [file ijms-25-07045-s001.zip › Fig S1.tif]
